# The frequency and impact of admission hyperglycemia on short term outcome of acute stroke patients admitted to Tikur Anbessa Specialized hospital, Addis Ababa, Ethiopia: a cross-sectional study

**DOI:** 10.1186/s12883-019-1578-x

**Published:** 2019-12-27

**Authors:** Yared Zenebe Zewde, Abenet Tafesse Mengesha, Yeweynhareg Feleke Gebreyes, Halvor Naess

**Affiliations:** 1 Department of Neurology, College of Health Sciences, Addis Ababa University, P.O.Box: 41690, Addis Ababa, Ethiopia; 2 Department of Internal Medicine, College of Health Sciences, Addis Ababa University, Addis Ababa, Ethiopia; 3 Department of Neurology, University of Bergen, Haukeland University Hospital, Bergen, Norway

**Keywords:** Hyperglycemia, Stroke severity, Functional outcome, Mortality, Africa

## Abstract

**Background:**

Admission hyperglycemia (HG) has been associated with worse outcomes among acute stroke patients. A better understanding and awareness of the potentially adverse influence of hyperglycemia on the clinical outcome of acute stroke patients would help to provide guidance for acute stroke management and prevention of its adverse outcomes. We aimed to assess the frequency of admission hyperglycemia and its impact on short term (30-days) morbidity and mortality outcomes of stroke in adult Ethiopian patients in an urban setting.

**Methods:**

A prospective, cross-sectional study was conducted among acute stroke patients admitted to Tikur Anbessa Specialized Hospital (TASH), within 72 h of symptom onset, from July to December 2016. Socio-demographic data, neuroimaging findings and capillary blood glucose values were obtained on admission. Hyperglycemia was defined as > 140 mg/dl. National Institute of Health Stroke Scale (NIHSS) and modified Rankin Scale (mRS) were used to assess the baseline stroke severity and the 30-days post-stroke outcome, respectively.

**Results:**

A total of 103 first-ever acute stroke patients were included (mean age = 55.5 + 15.3 years, 64.1% male and 65% under the age of 65 years) and 51 (49.5%) were hyperglycemic at time of admission. The median admission NIHSS score was worse in the hyperglycemic patients 14 (IQR 10–19) compared to normoglycemic patients 11 (IQR 8–15). Among stroke survivors, patients with hyperglycemia were 3.83 times (95% CI, 1.99–6.19) more likely to be functionally impaired (mRS = 3–5) at 30-days compared to normoglycemic patients (*P* = 0.041).Older age (≥ 65 years) (*P* = 0.017) and stroke severity (NIHSS > 14) (*P* = 0.006) at admission were both significantly associated with poor functional recovery at 30-day. Among patients who died at 30-day, two-third (66.7%) were hyperglycemic but they failed to show any significant association.

**Conclusions:**

Hyperglycemia is prevalent among Ethiopian stroke patients at the time of presentation and it is associated with significantly poor functional recovery at 30^th^-day of follow up. This finding provides a rationale for achieving normal blood glucose in the course of acute stroke management which could have a favorable impact on the neurological outcome and quality of life for patients.

## Background

Stroke remains a major global health problem, ranking the second most common cause of death and the third cause of disability worldwide [1]. Report from the 2013 Global Burden of Disease study showed that the absolute numbers of people affected by stroke every year, and lived with the consequence of stroke or died from their stroke, had increased significantly between 1990 and 2013 around the world. In 2013 globally there were 25.7 million stroke survivors, 6.5 million deaths from stroke, 113 million disability-adjusted life years (DALYs) due to stroke and 10.3 million new strokes. It was also found that the prevalence for both ischemic and hemorrhagic stroke nearly doubled across the globe over this period [2].

Although stroke related death and DALY rates have declined in both developed and developing countries over the past several decades, there is a significant increase in the absolute numbers of stroke deaths among younger adults aged between 20 and 64 years in developing countries, where the preponderance of the burden of stroke continued to reside and contributing 75.2% of deaths from stroke and 81.0% of stroke-related DALYs [1, 2].

In the past few years, developing countries including Sub-Saharan Africa (SSA) are exhibiting rapid urbanization and epidemiological transition with a sharp rise in the population number and life expectancy as well as changing lifestyle and a dietary habit which will increase the risk of cardiovascular disease, particularly stroke [3]. This changes in combination with poor health care service and lack of government commitment will contribute to a looming epidemic of stroke in the SSA regions.

Stroke patient’s outcome is influenced by a myriad of factors, including stroke subtype, stroke severity, associated comorbidities (prior history of diabetes mellitus (DM) or hypertension), post-stroke complications, and availability of acute stroke care facilities [4]. Admission hyperglycemia (HG) is one of the potentially modifiable factors associated with adverse stroke outcomes [5]. Several clinical and experimental studies have shown that admission hyperglycemia has a deleterious effect on short term outcomes of acute stroke patients, although some other studies have presented conflicting data [6, 7]. Elevated blood glucose during the acute phase of stroke is not always due to underlying diabetes mellitus and may be due to a stress-induced high cortisol response. However, the notion of causality from hyperglycemia or a consequence of more severe stroke remains controversial [6, 8].

There is a higher percentage of in-hospital case fatality and severe neurologic deficits among acute stroke patients admitted to tertiary care hospitals in Ethiopia as compared to the global average [9]. Little is known, however, about the relationship between admission hyperglycemia and stroke outcomes in SSA, particularly in the east Africa countries, where the number of neurologists is limited and access to stroke centers is negligible. So far there is no published data that has assessed the deleterious effect of high blood glucose on stroke patients’ outcomes in the region. The aim of this first prospective study among Ethiopian stroke patients was to assess the point-prevalence of admission hyperglycemia and its impact on short term neurological recovery and case fatality. We hypothesized that a high frequency of admission hyperglycemia and less favorable functional recovery with increased mortality rate would be seen in a cohort of Ethiopian stroke patients at thirty-days of follow up.

## Methods

### Study design

An institution based prospective, cross-sectional study was conducted at Tikur Anbessa Specialized Hospital (TASH) between July and December, 2016.

### Study setting

TASH is located in Addis Ababa, the capital of Ethiopia. It is the largest and the first tertiary level referral university teaching hospital with an estimated bed capacity of over 700. It has an emergency unit, an intensive care unit, a neurology ward, a clinical laboratory, and a radiology department with a Computerize Tomography (CT) and Magnetic Resonance Imaging (MRI) services. There is an integrated clinical service composed of highly trained neuro-radiologists, neurologists and emergency care physicians who serve for the entire nation.

### Study population

The study population was all patients presenting to the emergency department with neurologic deficits suggestive of acute stroke and who fulfilled the inclusion criteria of age greater than 14 years, presentation within 72 h of symptom onset, and confirmation of stroke diagnosis using emergency brain CT scan. A non-fasting capillary blood sample was used to estimate the admission blood glucose (BG) using a calibrated glucometer. Exclusion criteria were: age less than 14 years, patients with clinical course consistent with transient ischemic attack (TIA), trauma related cerebral hemorrhage, subarachnoid hemorrhage, prior neurologic deficit, prior stroke, unknown duration from symptom onset and who declined to give consent.

### Sample size and sampling technique

The sample size was calculated using the single population proportion formula n = Z *α* ^2^*pq/d^2^ = 164 with estimated population proportion of 30% [10], α = 5% and d = 5%. During the study period, all first-ever acute stroke patients who met the inclusion criteria were enrolled consecutively.

### Baseline data collection

The socio-demographic and clinical data were collected using a structured questionnaire that was pre-tested in 8 acute stroke patients having weakness on the non-dominant side and we got a complete response. We attempted to mitigate selection bias by randomizing participant selection using the same inclusion criteria detailed above. The National Institute of Health Stroke Scale (NIHSS) was used to measure the stroke severity. NIHSS is a 15-item neurologic examination stroke scale that sums the scores from individual elements to provide an overall stroke impairment score. It is a well-validated and commonly used assessment tool to evaluate the baseline neurologic deficit in acute stroke patients. The NIHSS score was dichotomized into: mild stroke (NIHSS score ≤ 14) and severe stroke (NIHSS score > 14) [11].

In all patients, BG was documented and hyperglycemia was defined as BG level ≥ 140 mg/dl and normoglycemia as BG < 140 mg/dl [12]. DM was diagnosed if the patient had a pre-existing diagnosis of DM or was taking anti-diabetics drugs before stroke onset. If the BG was > 200 mg/dl and a repeat Fasting blood glucose (FBG) test showed ≥126 mg/dl, then patients were classified as newly diagnosed DM based on ADA diagnostic criteria [13].

### Thirty-day outcome

The modified Rankin Scale (mRS) was used for the assessment of functional outcome at 30-day after the index stroke onset, which may coincide with the time of hospital discharge and for those who were discharged earlier, we followed them by telephone using standardized interview protocol. The mRS is six-point ordinal scale ranging from 0 (no symptoms) to 6 (death) measuring the degree of disability or dependence in everyday life, including instrumental and basic activities of daily living (ADL). The functional mRS scoring was subcategorized into; good outcome (independence in ADL, mRS score (0–2), and poor outcomes (dependence in ADL, mRS score 3–5). A mRS score of 6 signifies a patient death (30-day mortality) [14].

### Statistical analysis

Data were analyzed using the Statistical Package for Social Sciences version 20.0 software package (SPSS Inc., Chicago, IL, USA). Descriptive statistics were summarized using the frequency and proportion table for categorical data and mean (standard deviation) or median (interquartile range) for continuous data. The Pearson’s Chi square test were used to compare noncontinuous data. For dichotomous data, multiple logistic regression models were used to determine univariate and multivariate associations between 30-day outcomes and patient related variables including; demographic factors; prior history of DM; type of stroke based on neuroimaging findings; stroke severity by NIHSS and BG category. Adjusted odds ratios with 95% confidence interval (CI) were estimated. Level of statistical significance was set at *P* < 0.05.

### Ethics approval and consent to participate

Ethics approval was obtained from the Institutional Review Board (IRB) of College of Health Science in Addis Ababa University. A written informed consent was obtained from each patient when clinically appropriate. For patients with depressed consciousness or severe aphasia, written consent was obtained from their care takers. For participants aged 14–18 years, an assent form was obtained from their parents or legal guardians. Confidentiality and anonymity of all patient data were kept throughout the study. The study was performed in accordance with the Declaration of Helsinki.

## Results

### Socio-demographic characteristics

We approached 110 first-ever acute stroke patients, of whom 7 were excluded due to incomplete NIHSS data during hospitalization. The remaining 103 stroke patients were included in the final analysis having a mean age + SD of 55.5 + 15.3 years at presentation. The majority (64.1%) were male and around two-third (65%) were under the age of 65 years. Among our study participants, 82 (79.6%) were married and 8 (7.8%) were single. Thirty-two (31.1%) patients were farmers by occupation and 41 (39.8%) were illiterates with no formal education. Findings on initial brain CT scan classified stroke subtypes as 52 (50.5%) cases of ischemic and 51 (49.5%) hemorrhagic stroke cases. In 8 (7.8%) of the participants the diagnosis of diabetes was known before the stroke onset. While 7 of our patients had a BG > 200 mg/dl at presentation, three (2.9%) of them fulfilled the ADA diagnostic criteria [13] for a newly diagnosed DM after the stroke onset. Table 1 summarizes the socio-demographic and clinical characteristics of the participants.

**Table 1:** Socio-Demographic and Clinical Characteristics of acute stroke patients admitted to Tikur Anbessa Specialized Hospital in Addis Ababa, Ethiopia

Variables	Number	Percentage (%)
Age (mean + SD), years	55.5 + 15.3	
Age groups		
< 65 years	67	65.0
> 65 years	36	35.0
Gender		
Male	66	64.1
Female	37	35.9
Marital status		
Married	82	79.6
Widowed	13	12.6
Single	8	7.8
Educational status		
Illiterate	41	39.8
Elementary school	39	37.8
High school	13	12.6
College and above	10	9.7
Occupation		
Farmer	32	31.1
Merchant	27	26.2
Housewife	21	20.4
Government employee	15	14.5
Retired/Pension	4	3.9
Student	4	3.9
Type of stroke		
Ischemic stroke	52	50.5
Hemorrhagic stroke	51	49.5
Diabetes mellitus (DM)		
No DM diagnosis	92	89.3
Previously diagnosed DM	8	7.8
Newly diagnosed DM	3	2.9
Admission NIHSS score		
NIHSS ≤14	62	60.2
NIHSS > 14	41	39.8
mRS score at 30^th^ day		
mRS < 3	27	26.2
mRS ≥3	76	73.8

### Relationship between admission blood glucose level and clinical characteristics

Almost half (49.5%) of our study participants were hyperglycaemic at the time of admission with a BG level ≥ 140 mg/dl. The mean (+ SD) BG level in the normoglycemic and hyperglycemic groups were 119.9 + 13.0 mg/dl and 183.2 + 34.5 mg/dl, respectively. Admission hyperglycemic was presented in the majority 7/8 (87.5%) of diabetic patients and a multiple regression analysis showed that diabetes diagnosed before or after the stroke onset was related to high admission blood glucose level independent of age, sex, stroke subtype and baseline stroke severity (*P* = 0.023).

The median NIHSS score was higher in the hyperglycemic (BG) patients (14 [IQR 10–19]) as compared to the normoglycemic patients (11 [IQR 8–15]). But a multiple regression analysis showed there was no relationship between BG and stroke severity on admission NIHSS independent of age, sex, diagnosis of diabetes and stroke subtype (*P* = 0.42).

Among the study participants, hemorrhagic stroke was significant increased in patients with admission hyperglycemia (*n* = 33 (64.7%) vs *n* = 18 (35.3%); *P* = 0.002) when compared to those with normal blood glucose level on admission. The association between admission blood glucose and clinical characteristics of the participants are shown in Table 2.

**Table 2:** Factors associated with development of hyperglycemia at time of admission among acute stroke patients admitted to Tikur Anbessa Specialized Hospital in Addis Ababa, Ethiopia

Variable	Hyperglycemia (N = 51), (%)	Normoglycemia (N = 52) (%)	Adjusted OR (95% CI)	*P*-value
Age				
< 65 years	32 (47.7)	35 (52.3)	1.37 (0.52–3.62)	0.53
≥ 65 years	19 (52.7)	17 (47.3)	1	
Gender				
Male	33 (50.0)	33 (50.0)	0.83 (0.33–2.06)	0.68
Female	18 (48.6)	19 (51.4)	1	
Diagnosis of diabetes mellitus				
Yes	7 (87.5)	1 (12.5)	0.07 (0.01–0.69)	0.023
No	44 (46.3)	51 (53.7)	1	
Type of stroke				
Ischemic	18 (34.6)	34 (65.4)	1	
Hemorrhagic	33 (64.7)	18 (35.3)	4.24 (1.67–10.76)	0.002
Admission NIHSS score				
NIHSS > 14	25 (61.0)	16 (39.0)	1.53 (0.55–4.21)	0.42
NIHSS ≤ 14	26 (41.9)	36 (58.1)	1	
Median admission NIHSS (IQR)	14 (10–19)	11 (8–15)		
Mean BG + SD (mg/dl)	183.2 + 34.5	119.9 + 13.0		
Mean mRS + SD	3.82 + 1.34	3.33 + 1.52		

### Thirty-day functional recovery and mortality in relation to hyperglycemia and clinical characteristics

Among our stroke cohorts, the mean mRS score at 30-day was 3.6 + 1.45. Ninety-one patients (88.3%) had survived until 30th-day and majority (70.3%) of them showed poor functional recovery (mRS 3–5), while 27 (29.7%) patients had a good functional outcome (mRS 0–2). The association between clinical characteristics and 30-day functional outcome among survivors are shown in Table 3.

**Table 3:** Factors associated with 30th day poor functional outcome among stroke survivors who were admitted to Tikur Anbessa Specialized Hospital in Addis Ababa, Ethiopia

Variables	30th day functional outcome among survivors n = 91		Adjusted OR (95% CI)	*P*-value
	mRS 0–2 (n = 27,29.7%)	mRS 3–5 (n = 64,70.3%)		
Age				
< 65 years	22 (81.5)	34 (60.7)	1	
≥ 65 years	5 (14.3)	30 (85.7)	5.53 (1.36–22.43)	0.017
Gender				
Male	17 (29.3)	41(70.7)	0.99 (0.34–2.88)	0.98
Female	10 (30.3)	23 (69.7)	1	
History of diabetes mellitus				
Yes	1 (12.5)	7 (87.5)	0.29 (0.02–3.97)	0.35
No	26 (31.3)	57 (68.7)	1	
Admission Blood glucose level				
≥ 140 mg/dl	9 (20.9)	34 (79.1)	3.83 (1.99–6.19)	0.041
< 140 mg/dl	18 (37.5)	30 (62.5)	1	
Type of stroke				
Ischemic	18 (36.0)	32 (64.0)	3.52 (0.96–12.94)	0.058
Hemorrhagic	9 (21.9)	32 (78.1)	1	
Admission NIHSS score				
NIHSS > 14	3 (9.7)	28 (90.3)	0.12 (0.03–0.54)	0.006
NIHSS ≤ 14	24 (40.0)	36 (60.0)		

The functional recovery of stroke survivors at 30-days were compared across the different clinical and laboratory profiles. Accordingly, a multivariate logistic regression analysis showed patients with high blood glucose level at admission were 3.83 times (95% CI, 1.99–6.19) more likely to be functionally impaired (mRS = 3–5) at 30^th^-day compared to those with normal glucose (*P* = 0.041). The other factors that were significantly associated with poor functional outcome were older age (≥ 65 years) at time of stroke onset (OR 5.53; 95% CI,1.36–22.43; *P* = 0.017**)** and severe stroke (NIHSS > 14) (OR 0.12; 95% CI, 0.03–0.54; *P* = 0.006) at admission.

The 30-day mortality rate was 11.7% with a mRS score of 6. Mortality was significantly increased in patients younger than 65 years of age at time of stroke onset (*P* = 0.04) and severe stroke **(**NIHSS > 14) at time of hospitalization (*P* = 0.004). Although 15.7% of hyperglycemic patients died at 30-day, on multiple logistic regression no significant difference was found from normoglycemic patients (*P* = 0.94), after adjusting for age, sex, stroke type and severity (see Table 4).

**Table 4:** Factors associated with 30 -day mortality among stroke patients admitted to Tikur Anbessa Specialized Hospital in Addis Ababa, Ethiopia

Variables	Patients’ condition at 30th day		Adjusted OR (95% CI)	*P*-value
	Died (mRS = 6) n = 12, 11.7%	Alive (mRS < 6) n = 91, 88.3%		
Age				
< 65 years	11 (16.4)	56 (83.6)	0.05 (0.003–0.83)	0.04
≥ 65 years	1 (2.8)	35 (97.2)	1	
Gender				
Male	8 (12.1)	58 (87.9)	1.1 (0.18–6.89)	0.92
Female	4 (10.8)	33 (89.2)	1	
Admission Blood glucose level				
≥ 140 mg/dl	8 (15.7)	43 (84.3)	0.96 (0.15–6.36)	0.97
< 140 mg/dl	4 (7.7)	48 (92.3)	1	
Type of stroke				
Ischemic	2 (3.8)	50 (96.2)	1	
Hemorrhagic	10 (19.6)	41 (80.4)	4.62 (0.34–63.15)	0.25
Admission NIHSS score				
NIHSS > 14	10 (24.4)	31 (75.6)	0.039 (0.004–0.38)	0.004
NIHSS ≤ 14	2 (3.2)	60 (96.8)	1	

## Discussion

We performed this prospective cross-sectional study to determine the frequency of hyperglycemia and its effect on the short-term outcome of acute stroke patients in a resource limited setting where there are neither stroke units nor comprehensive rehabilitation centers exist.

The majority of our participants were males with M:F ratio of 1.8:1, and two-third of them were younger than 65 years with the mean age of 55.5 + 15.3 years at presentation. This is similar to previous studies in Ethiopia [15] and other studies in Africa [16, 17]. However, the average age of our stroke patients was younger than stroke victims in western countries. The median age reported by the European Registers of Stroke was 73 years [IQR 62 to 81] [18].

We found an equal proportion of ischemic (50.5%) and hemorrhagic strokes (49.5%) with 1.02:1 ratio. However, a higher proportion of ischemic stroke had been reported by other African countries [16, 17] in accordance with the western epidemiology [19]. Our result is similar to prior hospital-based studies in Ethiopia (1.72:1) [15] and (1.01:1) [20]. The possible explanation for the equal proportion of ischemic and hemorrhagic stroke in our setup may be related to the study setting, where most minor ischemic strokes and TIA’s were treated at the primary care centers with spontaneous resolution of clinical symptoms and minor disabilities that did not necessitate referrals to a tertiary care center like ours.

We have shown a high prevalence (49.5%) of hyperglycaemia in patients presenting with acute stroke to a hospital. This observation is consistent with several other studies in which the reported prevalence of admission hyperglycemia in acute stroke patients varied from 20 to 50%, depending on the study design (prospective or retrospective), the cut off value for hyperglycemia and stroke subtype [12, 21]. A 28% prevalence rate was reported by Ogunrin et al. [22], using the same hyperglycemic definition (≥140 mg/dl) from west Africa, Nigeria. But the authors attributed the lower prevalence to the retrospective design of their study and that might have affected their results, as only 100 of their 163 patients had blood glucose estimated at admission.

Diabetes mellitus is a well-recognized risk factor for stroke and the frequency of diabetes in our study was 10.7%. This finding is slightly higher than the prevalence of DM across different localities of Ethiopia, 0.3 to 7.0% [23]. We found a 2.9% prevalence of newly diagnosed diabetes which probably reflect the prevalence of undiagnosed diabetes in acute stroke population at time of hospitalization, although geographical variations noted. A comparable frequency of 5.3% was reported by Woo J and his colleagues, from a regional general hospital in Hong Kong [24]. While a higher 11% prevalence was reported by L.S. Williams et al. [25]. A study from USA showed a short median time between the onset of diabetes and stroke and a high incidence of newly-diagnosed diabetes in acute stroke patients could be expected [26]. These findings support the importance of screening all acute stroke patients for undetected diabetes and confirm the diagnosis by determining the fasting plasma glucose or glycated hemoglobin (HbA1c) level, especially if the admission glucose level was very high (> 200 mg/dl).

In this study, the median NIHSS score at admission was higher (14) in the hyperglycemic group as compared to (11) normoglycemic group. But on multiple logistic regression, we found no significant difference on stroke severity (NIHSS > 14) between the two groups after adjusting for age, sex, diabetes history and stroke type. In the contrary, Stead and his colleagues reported strong association between hyperglycemia and sever stroke at presentation [27].

Our patients with hyperglycemia had a significant disability with poor functional recovery (mRS ≥3) at 30-day. This finding is in line with hospital-based report among ischemic stroke patients in China, where hyperglycemia was identified as an independent predictor for a poor functional outcome (defined as mRS ≥3), at 30 and 90 days after the stroke onset [28]. Sun et al. [29] analyzed a large cohort of patients with intracerebral hemorrhage, which showed an elevated admission blood glucose was an independent predictor of 3-month poor outcome and they concluded that hyperglycemia has a greater prognostic value in non-diabetics than diabetics with a similar glucose level. A possible explanation for the negative association between hyperglycemia and poor functional outcomes after a stroke includes direct toxic effect of high blood glucose to ischemic neurons which may disrupt the blood-brain barrier and promote hemorrhagic infarct conversion [28] and also increasing superoxide production may exacerbate perihematomal cell death in the brain [29].

Stroke severity at admission was also significantly associated with poor functional outcome and increased risk of death at 30 days. Green SR et al. [8] compared 103 acute ischemic stroke patients based on their admission blood glucose level and they found a similar association, where functional outcomes at 30- and 90-day were poor among the diabetics and stress hyperglycemic groups who also had a higher NIHSS score of > 13 at admission These findings possibly explained by the prolonged hospital stay, severe motor weakness and a higher rates of medical complications that follows severe stroke.

Multiple studies [6, 12, 29] showed the relationship between high admission blood glucose and short term mortality among acute stroke patients at different time periods after stroke onset. However, in this study majority (66.7%) of the patients died at 30-day were hyperglycemic at admission but they failed to show the expected negative associations. This discrepancy could be explained by the small number of participants and those patients with severer stoke were possibly died before reaching the hospital. Several limitations must be acknowledged. First, the diagnosis of DM was based only on medical. history and FBG test where confirmatory tests like HbA1c were not done to exclude stress hyperglycemia. Second, a single-center, hospital-based study in an urban setting might introduce. selection bias and likely to overrepresent the more severe stroke cases referred for tertiary care. Therefore, we acknowledge the limited generalizability of our findings to the general population.

## Conclusion

Our analysis showed a higher prevalence of hyperglycemia in Ethiopian acute stroke patients at presentation and it had a significant association with poor functional recovery at short-term. The negative impact of admission hyperglycemia on functional recovery suggests that achieving normal blood glucose in the early stages of stroke may favorably influence the clinical recovery and quality of life of the patients. Further studies with a larger cohort in a multi-center setting are required to determine the independent role of hyperglycemia in neurological outcomes in stroke patients.

## Data Availability

The datasets used and/or analysed during the current study are available from the corresponding author on reasonable request.

## Abbreviations

ADA: American Diabetes Association
ADL: Activities of daily living
BG: Admission hyperglycemia
CT: Computerized Tomography
DALY’s: Disability-adjusted life years
FBG: Fasting blood glucose
GBD: Global Burden of Diseases
HbA1c: glycated hemoglobin
MRI: Magnetic resonance imaging
mRS: modified Rankin Scale
NIHSS: National Institute of Health Stroke Scale
SSA: Sub-Saharan Africa
TIA: Transient Ischemic Attack
